# Supplementary material for: Analysis Pipeline for Extracting Features of Cortical Slow Oscillations
Source: Front Syst Neurosci. 2019 Nov 22;13:70. doi: 10.3389/fnsys.2019.00070 (PMC6882866; doi:10.3389/fnsys.2019.00070)
Supplement: Supplementary file 1 [file Data_Sheet_1.PDF]

## Supplementary Figures

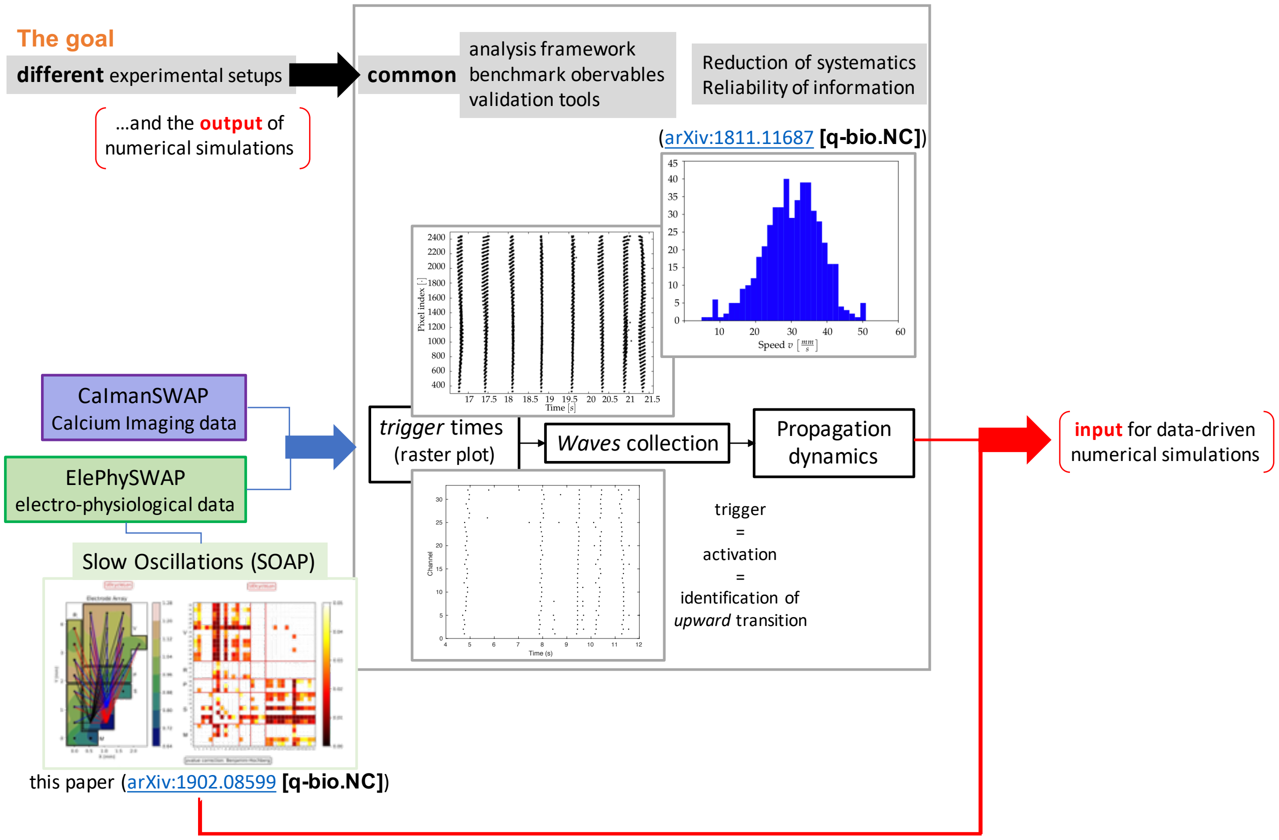


S-Figure 1. Schema of the full workflow. The idea behind the approach is: [phase 1] develop a strategy for extracting the "trigger times" (i.e. times of transition) from the raw data; for ECoG data, the method is based on the estimation of the multi-unit activity (MUA); [phase 2] once that the transition times are identified for each channel (electrode or pixel), the collection of triggers is sliced into "candidate waves"; the process is refined iteratively, up to obtaining a solid "waves collection", that enables systematics studies of the propagation of delta rhythms across the surface. In addition to the efforts devoted to the validation and improvement of phase 1 for ECoG data, the study presented in this work can be intended as a branch of the topic, focused on the local analysis of slow oscillations (SO), preliminary to the evaluation of spatio-temporal large-scale correlations highlighted in slow waves propagation. Indeed, for the EcoG data, the high temporal resolution of the recordings allows for the investigation of the basic temporal features, adding informative content to outcomes enabled with phase 2.

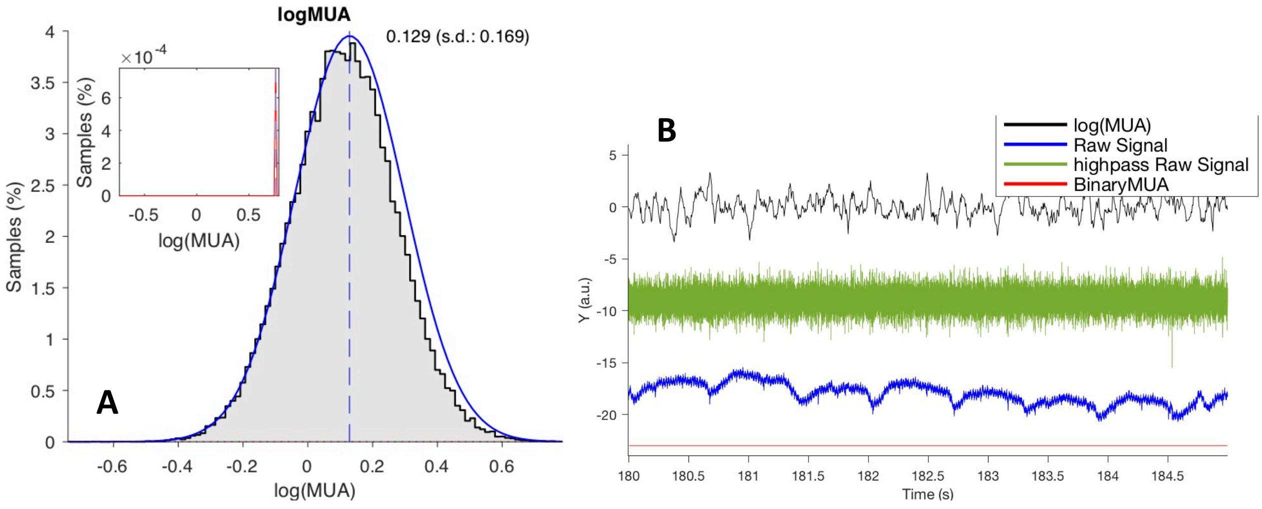


S-Figure 2. [Data File: 02; Channel: 31] → excluded for a failure in the DAQ. Indeed, the distribution of log(MUA) is monomodal, with no content associated with the "On" state. As a consequence, no transitions are identified (flat red line in Panel B), the *FewTransitions* alarm rings and the channel is excluded from further analysis. Different from the corresponding panel in Figure 4 in the manuscript, here Panel A contains the distribution of log(MUA) before it is shifted at the position of the peak, with the results of the Gaussian fit (mean and standard deviation) printed on the plot (see Figure 4, caption). The blue line is the fit curve, please note that the fit is executed on the left profile of the peak only.

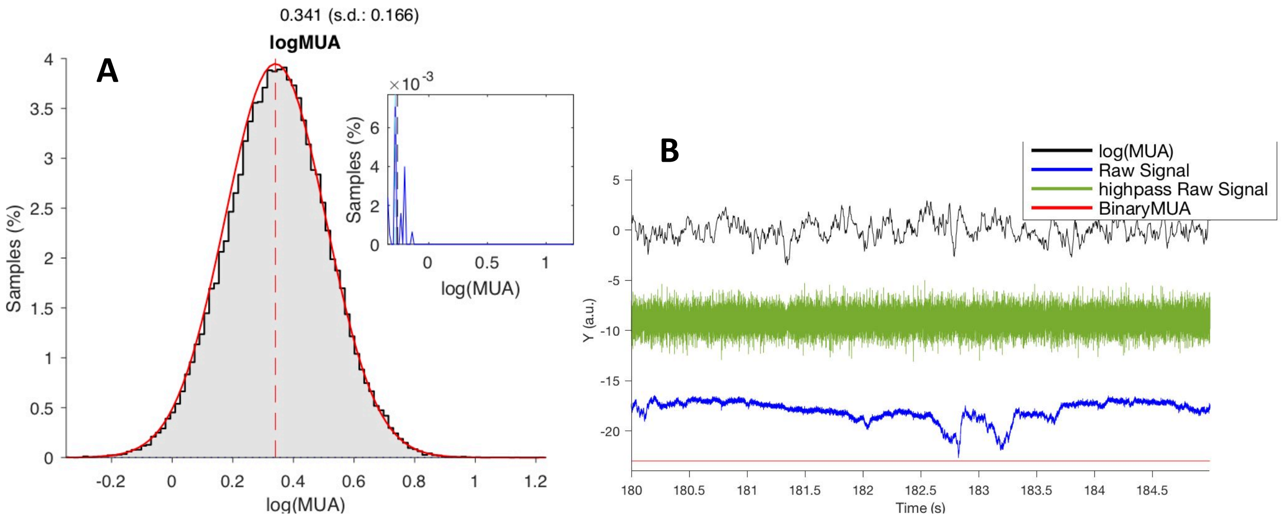


S-Figure 3. [Data File: 20; Channel: 01] → excluded for a negative asymmetry (*RightPeak* alert). Indeed, also here the distribution of log(MUA) is monomodal; the fit line is red (and the tail is blue, on the left) because the peak is found on the right of the range segment (in details: the algorithm checks if the position of the dominant peak is closer to the left (minimum) or to the right (maximum) value of the range of log(MUA) values). As in the previous case, no transitions are identified (flat red line in Panel B), the *FewTransitions* alarm rings and the channel is excluded. Panel A contains the distribution of log(MUA) before it is shifted at the position of the peak, with the results of the Gaussian fit (mean and standard deviation) printed on the plot (see Figure 4, caption). Please note that the fit is executed on the right profile of the peak only.

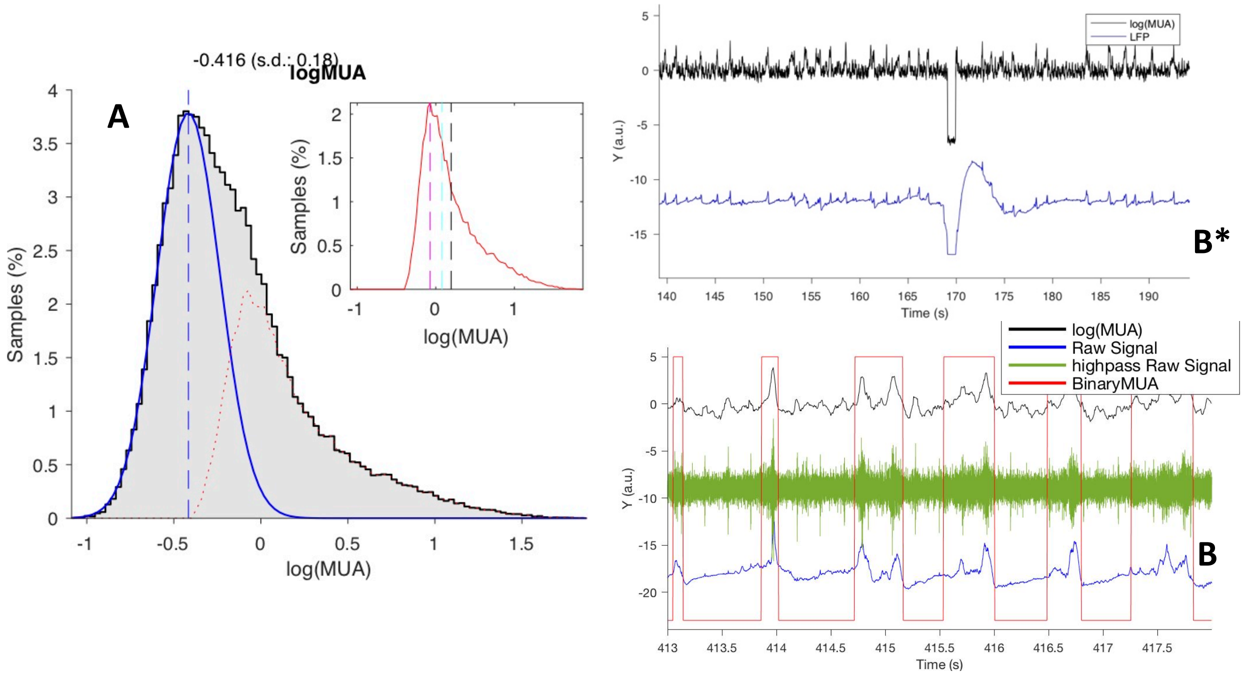


S-Figure 4. [Data File: 03; Channel: 04] → Panel B* shows that a discontinuity is found by the pipeline after about 170 s from the start of the recording (print out in the log is: "DISCONTINUITY FOUND 168.9911 170.0394", the time interval corresponding to the discontinuity is printed out). As discussed in Section 2.1 in the caption of Table 1, a discontinuity in the data acquisition that invests only a limited portion of time is a non-blocking condition, since it can be isolated and the channel can be recovered for the analysis, as can be seen here in Panels A and B, that present a behaviour comparable with what displayed in Figure 4 of the manuscript. Please note that here the content of the tail (the area below the red curve in Panel A) is larger than in Figure 4 Panel A, denoting a larger portion of time in the "On" state, in agreement to what in evidence in Panel B for the "Binary MUA" (red track) that shows more Up states in the same time interval, coherent with results on the frequency reported in Figure 8 in which experiment 03 manifests a larger mean frequency than experiment 01 (Figure 4 in the manuscript is obtained from Data File 01, Channel 01).

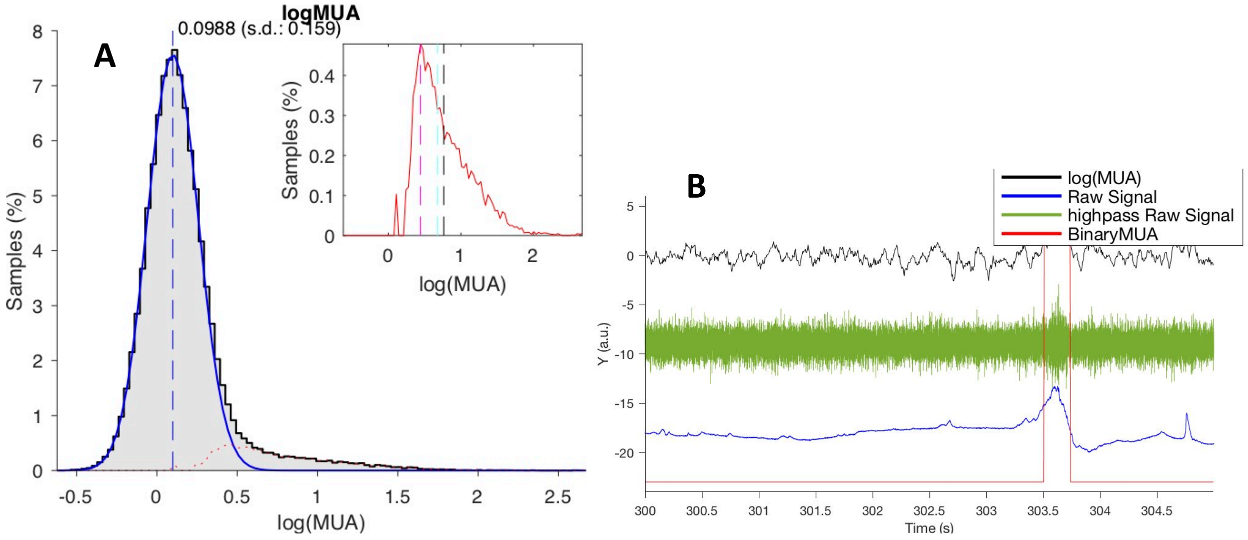


S-Figure 5. [Data File: 09; Channel: 17] → This channel results in a "WeakBimodality" alert, by which we intend that the area below the tail is lower than a given threshold (currently, equal to the 10% of the total area). The consequence is opposite to the case illustrated in S-Figure 4, the channel is "On" for a smaller portion of time, as in evidence looking at the BinaryMUA in Panel B. Please note that this is coherent with results of Figure 8, where experiment 09 has a lower mean frequency than experiment 01.

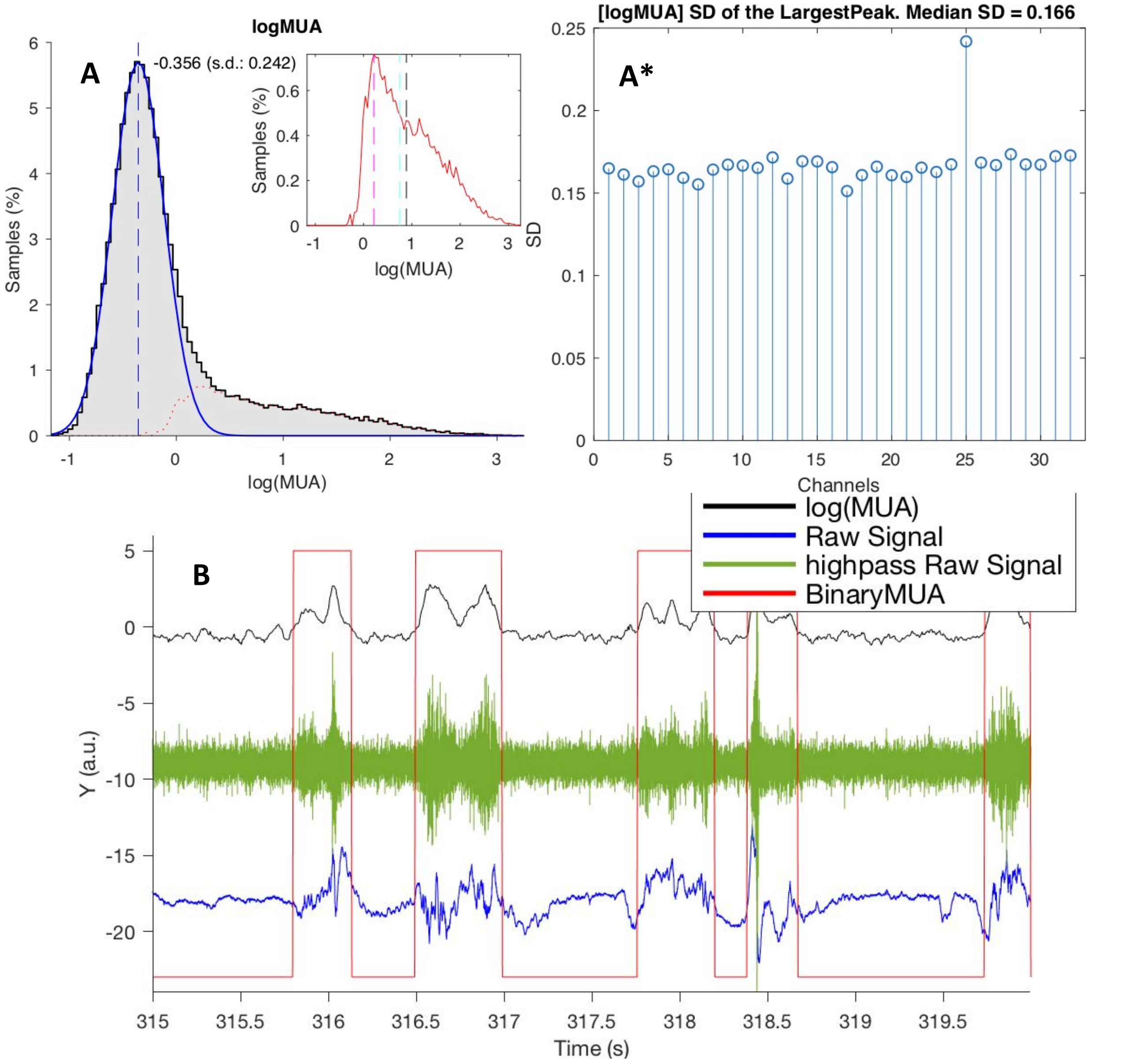


S-Figure 6. [Data File: 15; Channel: 25] → This channel is excluded because identified as "SD oulier" (see Table 1), according to the criterion of stability of the Gaussian peak. The channel appears as "standard" if looking at panel A and B, apart from the fact that the width of the peak in panel A (i.e. the sigma parameter of the fit) is larger than in other channels. The anomaly is identified at the end of the loop for the given experiment, when the statistics of the sigma parameters (SD) is evaluated, and a stem plot is produced (here identified as Panel A*).
